# Determinants of Stress Levels and Behavioral Reactions in Individuals With Affective or Anxiety Disorders During the COVID-19 Pandemic in Russia

**DOI:** 10.3389/fsoc.2022.870421

**Published:** 2022-07-05

**Authors:** Mikhail Yu Sorokin, Evgeny D. Kasyanov, Grigory V. Rukavishnikov, Maria A. Khobeysh, Olga V. Makarevich, Nikolay G. Neznanov, Tatyana G. Maximova, Dmitry N. Verzilin, Natalia B. Lutova, Galina E. Mazo

**Affiliations:** ^1^Department of Integrative Pharmaco-Psychotherapy of Patients With Mental Disorders, V.M. Bekhterev National Medical Research Center for Psychiatry and Neurology, Saint Petersburg, Russia; ^2^Department of Translational Psychiatry, V.M. Bekhterev National Medical Research Center for Psychiatry and Neurology, Saint Petersburg, Russia; ^3^Department of Geriatric Psychiatry, V.M. Bekhterev National Medical Research Center for Psychiatry and Neurology, Saint Petersburg, Russia; ^4^Department of Psychiatry and Addictions, I.P. Pavlov First Saint Petersburg State Medical University, Saint Petersburg, Russia; ^5^Faculty of Infocommunication Technologies, ITMO University, Saint Petersburg, Russia; ^6^St. Petersburg Institute for Informatics and Automation of the Russian Academy of Sciences, St. Petersburg Federal Research Center of the Russian Academy of Sciences, Saint Petersburg, Russia; ^7^Department of Management and Economy of Sports, Lesgaft National State University of Physical Education, Sport and Health, Saint Petersburg, Russia

**Keywords:** SARS-CoV-2, anxiety disorders, depressive disorder, bipolar disorder, psychological distress, health risk behavior

## Abstract

**Introduction:**

Individuals with affective and anxiety disorders are among those most vulnerable to the negative effects of the COVID-19 pandemic.

**Aim:**

This study aims to analyze the determinants of stress levels and protective behavioral strategies associated with the COVID-19 pandemic in Russian-speaking people with affective or anxiety disorders (AADs).

**Materials and Methods:**

In this cross-sectional online survey, the psychological distress and behavioral patterns of respondents with self-reported AAD (*n* = 1,375) and without disorders (*n* = 4,278) were evaluated during three periods of restrictive measures in Russia (March–May 2020). Distress levels were verified using the Psychological Stress Measure (PSM-25).

**Results:**

Stress levels among respondents with AAD were higher at all study periods than for those with no mental disorder (Cohen's *d* 0.8–1.6). The stress level increased (Cohen's *d* = 0.4) in adolescents (16–18 years) with AAD and remained the same in those without disorders; in youths (19–24 years) with and without disorders, an increase (Cohen's *d* = 0.3) and a decrease (Cohen's *d* = 0.3) in the stress were observed, correspondingly; the stress in adults (25–44 years) with disorders did not change and decreased in those without disorders (Cohen's *d* = 0.4). Individuals with bipolar disorders demonstrated lower stress than individuals with depressive (Cohen's *d* = 0.15) and anxiety disorders (Cohen's *d* = 0.27). Respondents with depressive and bipolar disorders employed fewer protective measures simultaneously and were less likely to search for information about COVID-19.

**Conclusion:**

The presence of affective or anxiety disorders is associated with a more acute response to the COVID-19 pandemic. Apparently, the type of mental disorder influenced stress levels and protective behavior patterns.

## Introduction

Stress associated with the COVID-19 pandemic has a complex multifactorial nature and an ambiguous profile of the behavioral reactions of the population (Fountoulakis et al., 2022). The danger of coronavirus infection has caused a wide range of psychological problems among the population of countries with high viral infection rates (Qiu et al., 2020). The greatest negative impact on mental health has been caused by such factors as: an unprecedented, potentially life-threatening situation of uncertain duration and economic consequences; increased family conflicts during large-scale quarantine measures in all major cities; an inconsistent information background with an oversupply of contradictory data (Sorokin et al., 2021; Vrublevska et al., 2021). The mental health consequences of such a crisis, including an increase in suicide rates, are predicted to continue for a long period of time and to peak after the actual pandemic (Pirkis et al., 2021).

Initial results confirmed that individuals with affective disorders are exposed to higher levels of stress, which in turn are associated with maladaptive situational and lifestyle changes occurring in response to the COVID-19 pandemic (Van Rheenen et al., 2020). In such individuals, the maladaptation and levels of preexisting anxiety and depressive symptoms are likely to increase with each subsequent wave of COVID-19 infection because they are more vulnerable to biological, social, and economic disruptions (Dabrowska et al., 2021). Moreover, individuals with affective or anxiety disorders are in high need of many variable factors associated with proper mental health care. Regular access to mental health-care services, medications, stable daily routines, and social interactions are necessary for those with mood illnesses. The psycho-social stress and limited access to the abovementioned elements could significantly affect the anxiety and mood symptoms in individuals with mental disorders (Asmundson et al., 2022). Subsequently, it was found that individuals with affective disorders have an increased risk of COVID-19 infection, as well as an increased risk of hospitalization and death (Diez-Quevedo et al., 2021). Thus, the impact of the COVID-19 pandemic on mental health is not equal for all groups of the population, especially for persons with major psychiatric disorders. Therefore, these imbalances in response to stress associated with the COVID-19 pandemic require more detailed study, taking behavioral reactions and socio-demographic indicators into account.

The study hypothesis is that the presence of affective or anxiety disorders is associated with a more acute response to the COVID-19 pandemic and epidemiological restrictions.

The study aims to analyze the determinants of stress levels and protective behavioral strategies associated with the COVID-19 pandemic in Russian-speaking people with affective and anxiety disorders.

## Methods

The study data were obtained through an extensive online survey conducted among Russian-speaking respondents during the restrictive period introduced as a measure to prevent the spreading of coronavirus infection. The most significant parts of the sample were obtained for 3 periods:

• 30 March to 8 April 2020 (1st period)–introduction of the first restrictive measures in Russia due to the worsening of the epidemiological situation;
• 29 April to 8 May 2020 (2nd period)–final stage of restrictive measures;
• 9 May to 18 May 2020 (3rd period)–cancellation of federal restrictive measures, early days of the post-restriction period.

Participants in the research were invited to complete an anonymous questionnaire *via* Google Forms, which took about 15 min. The questionnaire was distributed *via* social networks and on the websites of public organizations and thematic communities (refer to Acknowledgments).

The inclusion criteria were the ability to read Russian and consent to the processing of personal data. The non-inclusion criteria were the absence of values for individual points of the survey when filling in the questionnaire.

The questionnaire was based on self-reports on the socio-demographic characteristics of respondents and their place of residence, as well as on self-reports of their health status. The questionnaire, which was distributed in communities of patients with mental disorders, included a question on the presence/absence of a diagnosed affective or anxiety disorder with the option of choosing one of the proposed diagnoses in the questionnaire: depressive disorder, bipolar affective disorder, generalized anxiety disorder, cyclothymia, or dysthymia.

All participants in the study were invited to select any of the proposed concerns about the COVID-19 pandemic and any of the preventative measures they had implemented. Original questionnaire items which were already used earlier (Sorokin et al., 2020) described 10 types of concerns associated with COVID-19 (contagiousness of the virus; risk of isolation; the absence of specific treatment for COVID-19; fear for self-life; risk to the lives and health of relatives; possible financial difficulties; severe social consequences; lack of safety equipment for sale; possible lack of medication for daily intake; and impossibility of traditional way of life) and six behavioral patterns of infection prevention (wearing a mask or respirator; use of antiseptics; hand washing; social distance; and self-isolation). The reliability of these two subsets of dichotomous questions was calculated with the Kuder–Richardson-20 test: for concerns−0.41, for preventative measures−0.6. The results reflected the diversity of emotional and behavioral reactions of respondents, so these levels were considered satisfactory. Individual respondents could also indicate how often they requested information about the pandemic during the last week ranked by eight degrees, ranging from “never” to “hourly”.

Psychological stress scale (PSM-25) is 8-point Likert scale (“not at all” to “greatly”) used Lemyre in 1990 to assess current stress levels. Translated and adapted version for the Russian-speaking population was used (Vodop'yanova, 2009). The integral indicator of psychological stress in it is the total score, varying between 25 and 200. It reflects the expression of emotional, cognitive, and somatic reactions through the indicators of three subscales identifying three levels of stress. A total of 6 of the 25 questions (nos. 2, 7, 9, 15, 16, and 22) on the psychological stress scale describing somatic stress reactions were evaluated separately. A high score–a sum higher than 155 points–indicates a state of maladaptation and the need for correction; a score of 154–100 points indicates an average level of stress; low–under 100 points–indicates a state of psychological adaptation to workloads. In this study, PSM-25 demonstrated excellent internal consistency with Cronbach's alpha 0.949.

The study design was controlled by the independent ethical committee (IRB registration number: ∋ κ-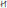-132/20). It was in conformity with the Declaration of Helsinki. It included a collection of anamnestic, socio-demographic data, and clinical parameters after the respondents signed a voluntary informed consent.

### Data Cleansing

We analyzed the values of the PSM-25 items to identify irrelevant answers and outliers. We used the scales of the PSM-25 items to calculate for all observations the Mahalanobis distances from the pattern consisting of average values. Then, we filtered out 11 outliers from the original 5,728 records. All outliers produced high Mahalanobis distances and revealed contradictory answers to interrelated questions. We also filtered out seven records with identical values in all PSM-25 items.

As there was no registration for the respondents, we checked the answers to the question: “Are you filling up this form for the first time?” For the repeated applications, we tried to find pairs with similar personal data as age, gender, educational level, marital status, occupation, and city. We identified 48 pairs (96 records) of repeat interviews of the same respondents. Among 48 pairs, we identified 26 where there was not <20 days between interviews. Those 26 pairs were analyzed separately as dependent samples. All 48 records of second interviews were removed from the main sample.

A total of three main grouping factors, including age, length of interview, and type of disorder (with no affective/anxiety disorder as a zero type), were used for extracting groups of records to be compared. We divided respondents into eight age groups and six periods. When comparing groups of records, we mostly used 1–5 age groups and 1–5 periods containing the majority of records.

### Exploratory Analysis

We used the ANOVA test, IBM SPSS Statistics (RRID:SCR_019096), to compare the amount and dynamic of distress in groups of respondents with/without affective or anxiety disorders. All groups corresponding to different time periods were separated. We obtained higher levels of distress for respondents with a disorder and different dynamics of distress levels for groups of respondents with/without a disorder (increase/reduction in the distress level).

We used regression analysis to examine whether the total distress level depended on age. For all groups of records, we observed negative dependency between these two variables. As the age of respondents was distributed rather differently in the groups under observation, we had to use more detailed analysis to distinguish the effects of disorder type and age on the distress level.

### Hypothesis Testing

When the gender composition of respondents was similar in all groups of observations (16% males and 84% females), the age distribution was essentially different. For example, the average age of respondents with a disorder was about 24, compared with 34 for those without a disorder.

For matching different groups of observations, we excluded random records, so that relative frequencies of ages became equal–not attempting to fit samples to an ideal, but filtering all the samples, so that the total number of records removed was minimal. We solved two optimization tasks: in the first task, we removed as few records as possible; in the second task, we used weights equal to inverse values of the sample sizes. The second task was used when the sample sizes were essentially different.

To compare different groups, we used factorial or one-way ANOVA and estimated standard errors and 95% confidential intervals for average values of dependent variables. We also performed *post hoc* analysis. When the variable did not match Gaussian distribution, we always used nonparametric tests, specifically repeated Mann–Whitney tests for two independent samples. However, we confirmed the fact that ANOVA tests are robust to the violation of normality for large sample sizes, as in our comparisons, ANOVA and nonparametric tests gave similar results. When testing hypothesis for all the PSM-25 items, we took into account multiple comparisons. However, there was no need to lower the level of significance, as *p*-values were usually low and there were many positive results among the PSM-25 items.

### Sampling Characteristics

Based on the self-report data on the presence of mental disorders, the final sample of 5,662 records was divided into two groups. The research group included 1,375 records (24.1%) containing information on the presence of affective pathology: 590 (10.3%) depressive disorders (including dysthymia), 530 (9.3%) bipolar disorders (including cyclothymia), and 255 (4.5%) anxiety disorders (general anxiety disorder, and panic disorder). The control group included 4,278 respondents (75.9%) who reported no affective or anxiety disorders.

To assess the age differences, the following subgroups of respondents within the research and control groups were included in the analysis: adolescents from 16 to 18 (1.6 and 1.8%, respectively), young adults from 19 to 24 (2.5 and 4.1%, respectively), and adults from 25 to 44 (19.9 and 42.7%, respectively). In all the subgroups analyzed (age, history of diseases, and specificity of reactions to the pandemic), the male to female ratio in the sample remained stable: 16 and 84%, respectively.

The survey covered respondents living in all federal districts of Russia. Residents of major cities made up 19.2 and 35% of the sample (Moscow and St. Petersburg, with populations of over 10 million and 5 million, respectively). Residents of other cities with populations of over one million accounted for 16.2%. Respondents from cities with a population of less than one million people constituted 29.6% of the sample.

## Results

### Stress in Comparison Groups

**Figure 1:**
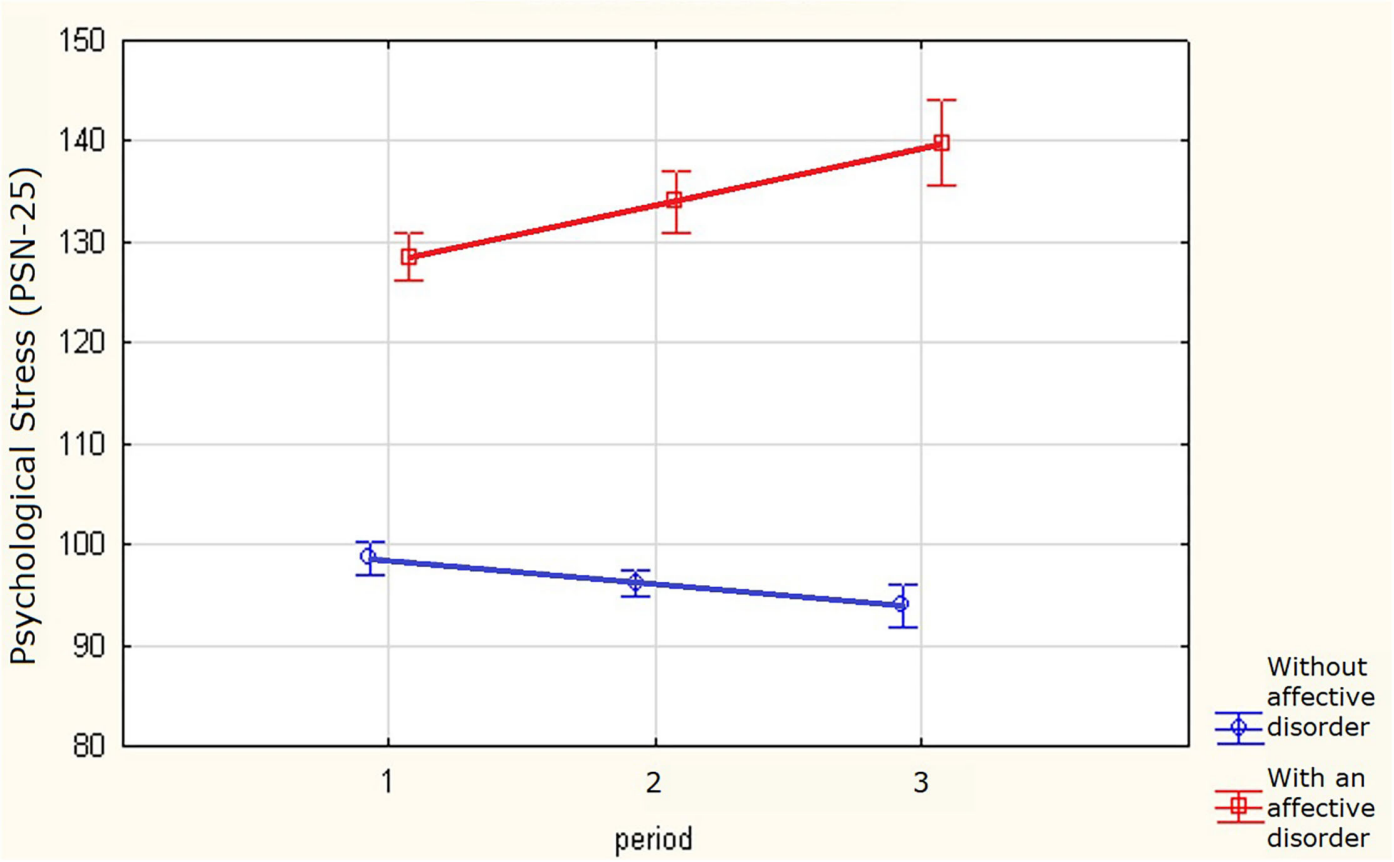
Levels and dynamics of stress for respondents with/without affective or anxiety disorders.

In the exploratory analysis, data were obtained on significantly higher rates of psychological stress (Cohen's *d* 0.8–1.6) in respondents with affective or anxiety disorders than for those with no mental disorder (Figure 1). At this point, we examined full groups of respondents with no adjustments to the age structures. In factorial ANOVA, we obtained significant differences with *p* < 2e-8 between groups for the factor of disorder (yes/no) and for the join factor disorder^*^period. We obtained *p*=0,051 for the factor of period. *Post hoc* analysis (least significant difference (LSD) test) confirmed the differences with *p* < 0,03 for all 2^*^3 = 6 groups except the pair period=2 and period=3 in the control group. For the factor of period, the tests of homogeneity of variances (Hartley F-max, Cohran C, Bartlett'sh chi-square) passed. The test failed for the factor of disorder. However, we can assume that the difference between the groups of respondents with/without affective disorder is too high (*p* < 1e-15) to be overturned with homogeneity tests.

In all age subgroups and time periods, respondents self-reporting affective or anxiety disorders (research groups) continued to show significantly higher rates of psychological stress than those with no affective/anxiety disorders (control group). It is noteworthy that the differences in stress levels between the control and research groups in the overall sample increased from the introduction of epidemiological restrictions to the period after their cancellation. However, these dynamics were not uniform in individual age groups.

### Dynamics of Stress Levels Between Periods of Epidemiological Restrictions

**Figure 2:**
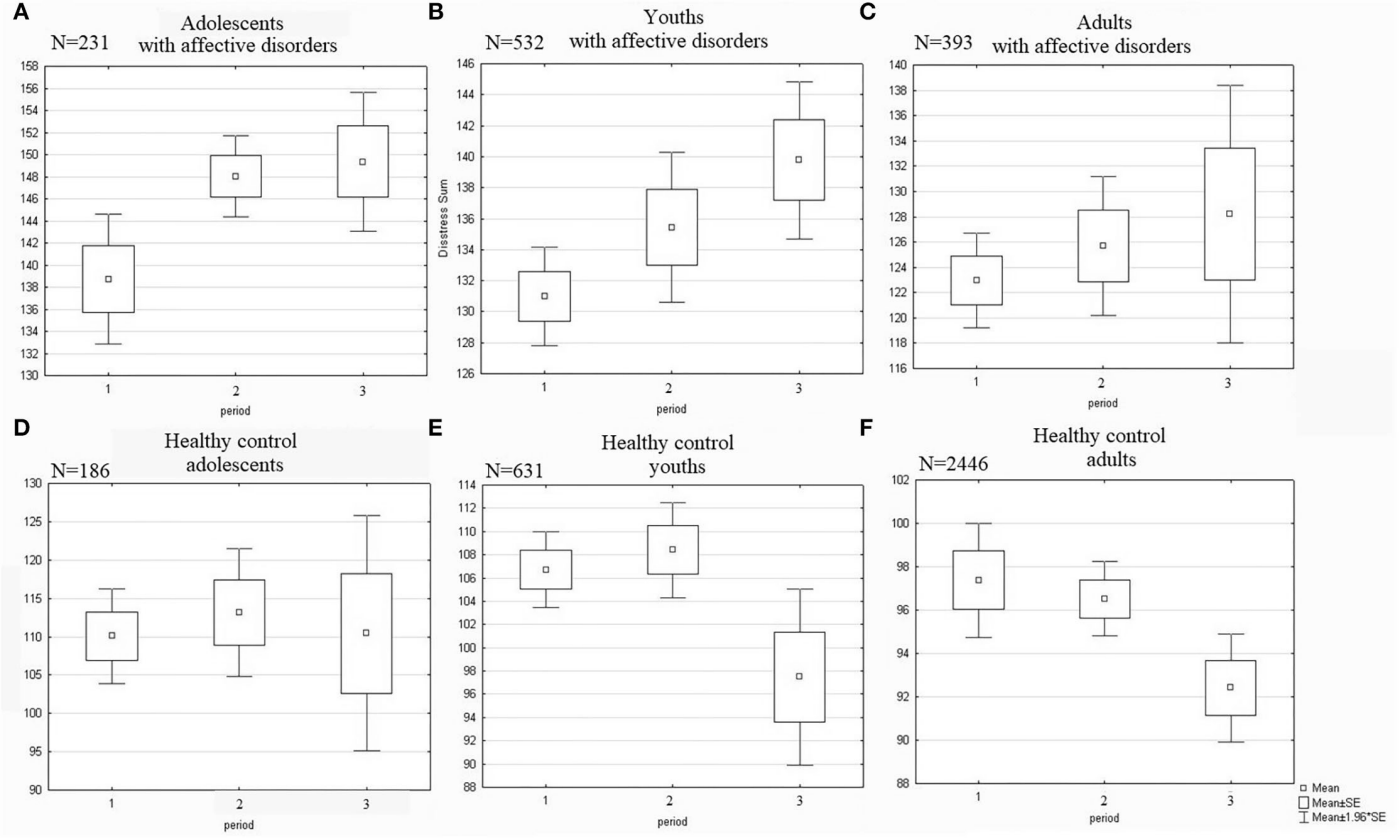
Dynamics of stress levels in adolescents and youths in contrast to adults. Significant differences **(A)** between “1” and “2” with *p* = 0.036. **(B)** between “1” and “3” with *p* = 0.02. **(E)** between “1” and “3” with *p* = 0.039 and between “2” and “3” with *p* = 0.012. **(F)** between “1” and “3” with *p* = 0.008 and between “2” and “3” with *p* = 0.017. No significant differences between periods in adults with affective disorders **(C)** and in healthy control adolescents **(D)**.

Among the three age subgroups, an increase in stress levels in the research group and a reduction in the control group between the 1st and 3rd periods were observed only among young adults aged 19–24 (Cohen's d=0.32 and Cohen's d=0.30; Figures 2B,E). In all the remaining figures, we performed the Mann–Whiney U test to confirm inter-group differences as all samples were rather far from normal distribution. Adolescents aged 16–18 from the research group showed higher rates of psychological stress in the 3rd period than those interviewed during the introduction of restrictive measures in the 1st period (Cohen's *d* =0.39, Figure 2A), but no reliable control dynamics were revealed (Figure 2D). Among adults in the control group, a reduction in stress levels between the 1st and 3rd periods was observed (Cohen's *d* = 0.40, Figure 2F), but there were no reliable dynamics in the research group (Figure 2C).

**Figure 3:**
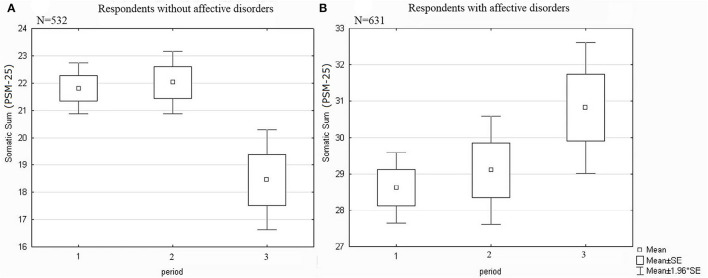
Dynamics of somatization in presence of an affective/anxiety disorder among young adults. Significant differences **(A)** between “1” and “3” with *p* = 0.01 and between “2” and “3” with *p* = 0.005. **(B)** between “1” and “3” with *p* = 0.046.

High levels of stress among young adults in the research group were associated with higher somatic rates on the PSM-25 scale in the 3rd period compared with the 1st period (Cohen's *d*=0.26, Figure 3A). In contrast, individuals aged from 19 to 24 in the control group who were examined after the removal of the anti-epidemic restrictions showed a lower level of somatization than those examined at the beginning of quarantine in the 1st period (Cohen's *d*=0.40, Figure 3B).

### Nosological Characteristics of Stress and Behavior Associated With the Pandemic

**Figure 4:**
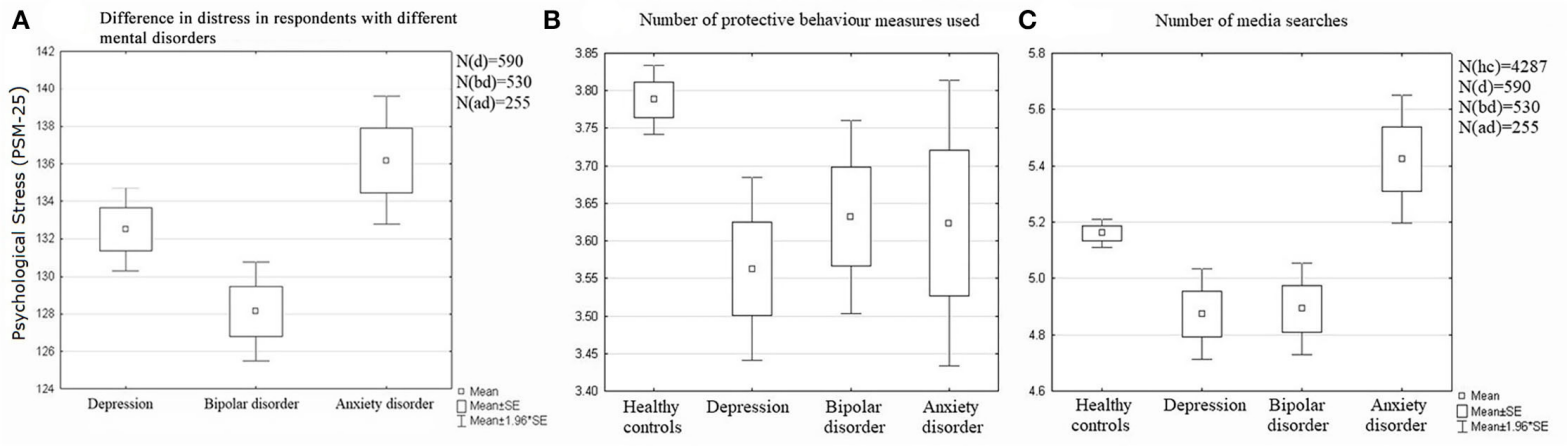
Stress levels, anxiety, and behavioral reactions in respondents depending on the presence of an effective anxiety disorder. Significant differences **(A)** between “bd” and “ad” with *p* = 0.002. **(B)** between “hc” and “d” with *p* = 0.0001 and between “hc” and “bd” with *p* = 0.043. **(C)** between “hc” and “d” with *p* = 0.017, between “hc” and “bd” with *p* = 0.012, between “hc” and “ad” with *p* = 0.001, between “d” and “ad” with *p* = 0.0002, and between “bd” and “ad” with *p* = 0.0001.

The level of stress on the PSM-25 scale was specifically associated with affective/anxiety disorders. Among subgroups of respondents with depressive, bipolar, and anxiety disorders, individuals with bipolar disorders demonstrated significantly lower levels of stress compared with individuals with depressive (Cohen's *d*=0.15) and anxiety disorders (Cohen's *d*=0.27) (Figure 4A).

It is also important to note that stress response characteristics were combined with the modification of protective behavior (Figure 4B) and the search for information about the pandemic (Figure 4C) both in the nosological subgroups of the research group and in the control group.

Respondents self-reporting depression and bipolar disorder used fewer protective measures simultaneously compared with the control group. However, there was a significant reduction in the concurrently practiced means of preventing infection only among those who reported depressive disorders (Cohen's *d* = 0.15), whereas among respondents with bipolar disorders the narrowing of protective measures were negligible (Cohen's *d* = 0.1). No reliable differences were found between the control group and the subgroup with anxiety disorders.

In the subgroup with depressive or bipolar disorders, respondents were less likely to search for news about the pandemic than those in the subgroup of anxiety disorders (Cohen's *d* = 0.28 and 0.28, respectively), and in comparison with the control group (Cohen's *d*=0.17 and 0.16, respectively). Participants self-reporting an anxiety disorder were the most likely to turn to the news (compared with depressive or bipolar disorders, Cohen's *d* = 0.28 and 0.28, respectively; with Cohen's *d* = 0.16). Respondents in the control group demonstrated an average frequency of searching for information about the pandemic.

## Discussion

Our research has demonstrated that the presence of affective or anxiety disorders is associated with a more severe response to the COVID-19 pandemic in different periods. Based on the socio-demographic characteristics, data on the behavioral reactions of the population and place of residence, as well as on the results of psychometric research on stress levels, we made four main observations.

First, stress levels among respondents self-reporting an affective or anxiety disorder were higher at all periods of the study than among those with no mental disorders. Second, the dynamics of stress levels in the research and control groups were heterogeneous and varied across the age subgroups. Third, the type of affective disorder influenced protective behavioral patterns and intensity of searching for information about the pandemic. Fourth, individuals with bipolar disorders had significantly lower stress levels than respondents with depressive or anxiety disorders.

As far as we can ascertain from available literature, this is the first study to provide evidence that multidirectional dynamics of stress during the COVID-19 pandemic are determined not only by the affective status of respondents but also by their age groups. In a sample of adolescents (16–18) and young adults (19–24) reporting a history of affective/anxiety disorders, average stress levels at the time of the cancellation of restrictive measures (period 3) were higher than at the time of the introduction of epidemiological restrictions (period 1). Among young and adult respondents who denied having mental disorders, stress levels at the final stage of the restrictive measures (period 2) were lower than those initially identified.

The differences in stress levels and their dynamics in respondents who confirmed or denied the presence of affective/anxiety disorders (taking nosology into account) were linked to their behavioral patterns. An increase in time spent searching for information about the pandemic is known to be directly associated with increased anxiety (Nekliudov et al., 2020). At the same time, the usage of hand hygiene can be associated with the reduction of anxiety and stress associated with COVID-19 (Wang et al., 2020). In our sample, the history of anxiety disorders was associated with frequent searching for news about the pandemic. At the same time, the history of bipolar or depressive disorders was associated with less searching for news about COVID-19 in the media. Most notable is that respondents who reported a history of depressive disorders practiced the fewest protective behavioral strategies. Thus, the relatively favorable course of stress reactions in respondents with a history of bipolar disorders, on the contrary, was linked to a slight reduction in their protective behavioral patterns in relation to coronavirus.

The differences identified in behavior associated with the search for information about COVID-19 and protective measures in respondents from different nosological groups may be seen as a predisposition for a more effective response to stress among respondents self-reporting a bipolar disorder and respondents without mental disorders and less effective response among respondents self-reporting depressive or anxiety disorders. The wider spread of pandemic anxiety known from bipolar disorder literature is unlikely to be associated with the development of severe distress in our sample (Van Rheenen et al., 2020). It is possible that a stressful response to the COVID-19 pandemic may be related not to the intensity of anxiety stress but to a disturbance of an individual's adaptive-compensatory reactions (Sorokin et al., 2021). The different results regarding bipolar disorders in our study and the COLLATE project can also be explained by the use of different psychometric tools (Van Rheenen et al., 2020).

According to our data, this is one of the largest studies of the determinants of stress levels in the Russian population, which took into account the presence of mental disorders. The results of this study formed the basis for the development of algorithms for the diagnosis and therapy of mental disorders registered during the COVID-19 pandemic in Russia (Neznanov et al., 2021). The findings are important for public health to take preventive screening measures among the population to reduce the burden of the COVID-19 pandemic.

### Limitations

The study had several limitations. First, it had a cross-sectional rather than longitudinal design, so the information on stress dynamics should be interpreted as a population change in response to the pandemic rather than as an increase or reduction in stress among the respondents over time. Second, data on the psychiatric condition of the subjects were based on their self-reports. According to the literature, this is strongly related to the results of medical history collection but does not enable us to speak about the verified diseases of respondents. Third, the need to comply with quarantine restrictions determined that the only possible format for conducting a study in the initial stages of the pandemic was in the form of an online questionnaire, which also had a number of features: the predominant participation of women in such studies and selection errors for persons who are not active users of the Internet. Fourth, the internal consistency of two subsets of questions about COVID-19 concerns and protective behavior was low. Meanwhile, according to Lee J. Cronbach, the reliability measure could reflect not only the consistency among items in a test but also the agreement among scorers of a performance test and the stability of performance of scores on multiple trials of the same procedure (Cronbach and Shavelson, 2004). In this sense, our results were taken into account as satisfactory and reflecting inter-subjects' diversity of COVID-19 reactions, as well as the differences revealed within periods of the pandemic and served an addition to main psychometric instrument (PSM-25) which demonstrated excellent reliability. Fifth, a number of data obtained in the course of the study, in particular about the specifics of somatic diseases of respondents, their education, family status, and the current level of the epidemic process in the region of their residence, were not taken into account in the analysis in this article, as they require further dynamic study taking into account the protracted nature of the pandemic.

## Conclusion

Assessment of the population's psychological reactions to the COVID-19 pandemic is a complex task that requires not only consideration of socio-geographical (age, residence) and clinical characteristics (history of affective or anxiety disorders), but also an analysis of the time periods. Individuals self-reporting affective or anxiety disorders tend to respond more emotionally to the pandemic by forming a wide range of anxiety concerns and make less effective use of protective behavioral strategies. As a result, this may determine different trends in stress response: an increase in distress during a pandemic among those who report affective/anxiety disorders and a reduction among those who report no mental disorders. Given the dynamics observed, psychiatric services should be prepared for a greater burden of affective and anxiety disorders after the actual end of the pandemic, especially among young people. Future studies should pay more attention to the secondary mental health effects of the COVID-19 pandemic on the most vulnerable groups.

## Data Availability Statement

The raw data supporting the conclusions of this article will be made available by the authors, without undue reservation.

## Ethics Statement

The studies involving human participants were reviewed and approved by Independent Ethics Committee in V. M. Bekhterev National Medical Research Center for Psychiatry and Neurology. The patients/participants provided their written informed consent to participate in this study.

## Author Contributions

Conceptualization of the study, goals, and aims: GM, NL, and EK. Investigation: EK, MS, OM, GR, and MK. Methodology and project administration: GM, NL, EK, and MS. Resources, writing, reviewing, and editing: NN, GM, and NL. Statistics: TM, DV, and MS. Writing (original draft): MS, EK, TM, and DV. All authors read and approved the final version of the manuscript.

## Conflict of Interest

The authors declare that the research was conducted in the absence of any commercial or financial relationships that could be construed as a potential conflict of interest.

## Publisher's Note

All claims expressed in this article are solely those of the authors and do not necessarily represent those of their affiliated organizations, or those of the publisher, the editors and the reviewers. Any product that may be evaluated in this article, or claim that may be made by its manufacturer, is not guaranteed or endorsed by the publisher.
